# Single port robot-assisted transperitoneal kidney transplant using the sp® surgical system in a pre-clinical model

**DOI:** 10.1590/S1677-5538.IBJU.2019.191

**Published:** 2020-08-10

**Authors:** Juan Garisto, Mohamed Eltemamy, Riccardo Bertolo, Eric Miller, Alvin Wee, Jihad Kaouk

**Affiliations:** 1 Department of Urology Cleveland Clinic ClevelandOhio United States Department of Urology, Cleveland Clinic, Cleveland, Ohio, United States;; 2 Cleveland Clinic Main Campus Hospital ClevelandOhio United States Cleveland Clinic Main Campus Hospital, Cleveland, Ohio, United States

## Abstract

**Introduction:**

Minimally invasive surgery has recently gained interest for kidney transplantation. We aimed to describe the step-by-step technique for single-port robotic transperitoneal kidney transplantation using the SP® surgical system (Intuitive Surgical, Sunnyvale, Ca) in a pre-clinical model.

**Materials and Methods:**

A male fresh cadaver model was placed in a lithotomy position. A 3cm midline incision was made 4cm cephalad to the belly button. An advanced access platform (GelPOINT, Rancho Margarita, California, USA) was inserted into the abdominal cavity through the incision. A left kidney was obtained for the local procurement organization. Bench preparation of the kidney was performed. Thereafter, the organ was introduced transperitoneal through the Alexis® wound retractor. The SP® robotic platform was docked and the pelvic fossa was targeted. The standardized steps of robotic multi-arm kidney transplant were duplicated. Primary outcomes such as intraoperative complications, rate of conversion to standard technique and operative times were recorded.

**Results:**

The procedure was technically completed using the SP® robotic system without conversion or the need for additional ports. There were no intraoperative complications. The total operative time was 182 minutes, with 35 minutes spent for bench kidney.

**Conclusions:**

Robotic Single-Port kidney transplantation using the SP® surgical platform is feasible in a pre-clinical model. The platform could be particularly interesting for multi-quadrant surgery such as auto-transplantation, potentially reducing the time for redocking. Further clinical studies in humans and comparison with standard surgical techniques are warranted.

